# Promising candidates for extracorporeal cardiopulmonary resuscitation for out-of-hospital cardiac arrest

**DOI:** 10.1038/s41598-020-79283-1

**Published:** 2020-12-17

**Authors:** Yo Sep Shin, Youn-Jung Kim, Seung Mok Ryoo, Chang Hwan Sohn, Shin Ahn, Dong Woo Seo, Won Young Kim

**Affiliations:** Department of Emergency Medicine, Asan Medical Center, University of Ulsan College of Medicine, 88 Olympic-ro 43-gil, Songpa-gu, Seoul, 05505 South Korea

**Keywords:** Cardiology, Medical research

## Abstract

Precise criteria for extracorporeal cardiopulmonary resuscitation (ECPR) are still lacking in patients with out-of-hospital cardiac arrest (OHCA). We aimed to investigate whether adopting our hypothesized criteria for ECPR to patients with refractory OHCA could benefit. This before-after study compared 4.5 years after implementation of ECPR for refractory OHCA patients who met our criteria (Jan, 2015 to May, 2019) and 4 years of undergoing conventional CPR (CCPR) prior to ECPR with patients who met the criteria (Jan, 2011 to Jan, 2014) in the emergency department. The primary and secondary outcomes were good neurologic outcome at 6-months and 1-month respectively, defined as 1 or 2 on the Cerebral Performance Category score. A total of 70 patients (40 with CCPR and 30 with ECPR) were included. For a good neurologic status at 6-months and 1-month, patients with ECPR (33.3%, 26.7%) were superior to those with CCPR (5.0%, 5.0%) (all *P*s < 0.05). Among patients with ECPR, a group with a good neurologic status showed shorter low-flow time, longer extracorporeal membrane oxygenation duration and hospital stays, and lower epinephrine doses used (all *P*s < 0.05). The application of the detailed indication before initiating ECPR appears to increase a good neurologic outcome rate.

## Introduction

Despite advances in cardiopulmonary resuscitation and post-cardiac arrest care, mortality and neurologic morbidity in patients with out-of-hospital cardiac arrest (OHCA), has remained poor^1^. The implementation of extracorporeal membrane oxygenation (ECMO) during ongoing cardiopulmonary resuscitation (CPR) has also been adopted for patients with OHCA, which is called extracorporeal cardiopulmonary resuscitation (ECPR)^2^. Several studies suggest ECPR for cardiac arrest is associated with improved survival ranging from 33 to 54% when there is a reversible cause for cardiac arrest, despite limited observational data in selected patient groups^3,4^. Given that two-thirds of OHCAs are caused by primary cardiac origin^5,6^, the initiation of ECPR for patients with refractory OHCA appears reasonable as it allows a second chance to maintain systemic circulation, and it provides patients with OHCA an opportunity to have several interventions proven to increase survival rates, such as percutaneous coronary intervention (PCI) and targeted temperature management, by identifying potential reversible causes of the cardiac arrest and administering treatment^7–9^. Current recommendations suggest using ECPR for select cardiac arrest patients who are refractory to initial conventional CPR (CCPR), which is usually 10 min^10–12^. However, ECPR is a complex process that requires a highly trained team, specialized equipment, and multidisciplinary support within the local healthcare system^13^. Therefore, broad, unselected application of ECPR should be avoided since only specific patients with OHCA might derive survival benefits from ECPR.

Although there is growing evidence that carefully selected patients with in-hospital cardiac arrest can benefit from organ perfusion, thereby potentially improving outcomes of in-hospital cardiac arrest, subsequent outcomes of ECPR in OHCA were inconsistent, showing a wide variation of survival rates^3,14,15^. Given the lack of clear guidelines for selecting patients for ECPR, the establishment of appropriate criteria for ECPR in the OHCA population is warranted. We, therefore, set our own criteria for implementing ECPR in patients with refractory OHCA based on previous studies and assessed the differences in good neurologic outcome and survival rates before and after application of the criteria.

## Results

### Baseline characteristics

**Figure 1:**
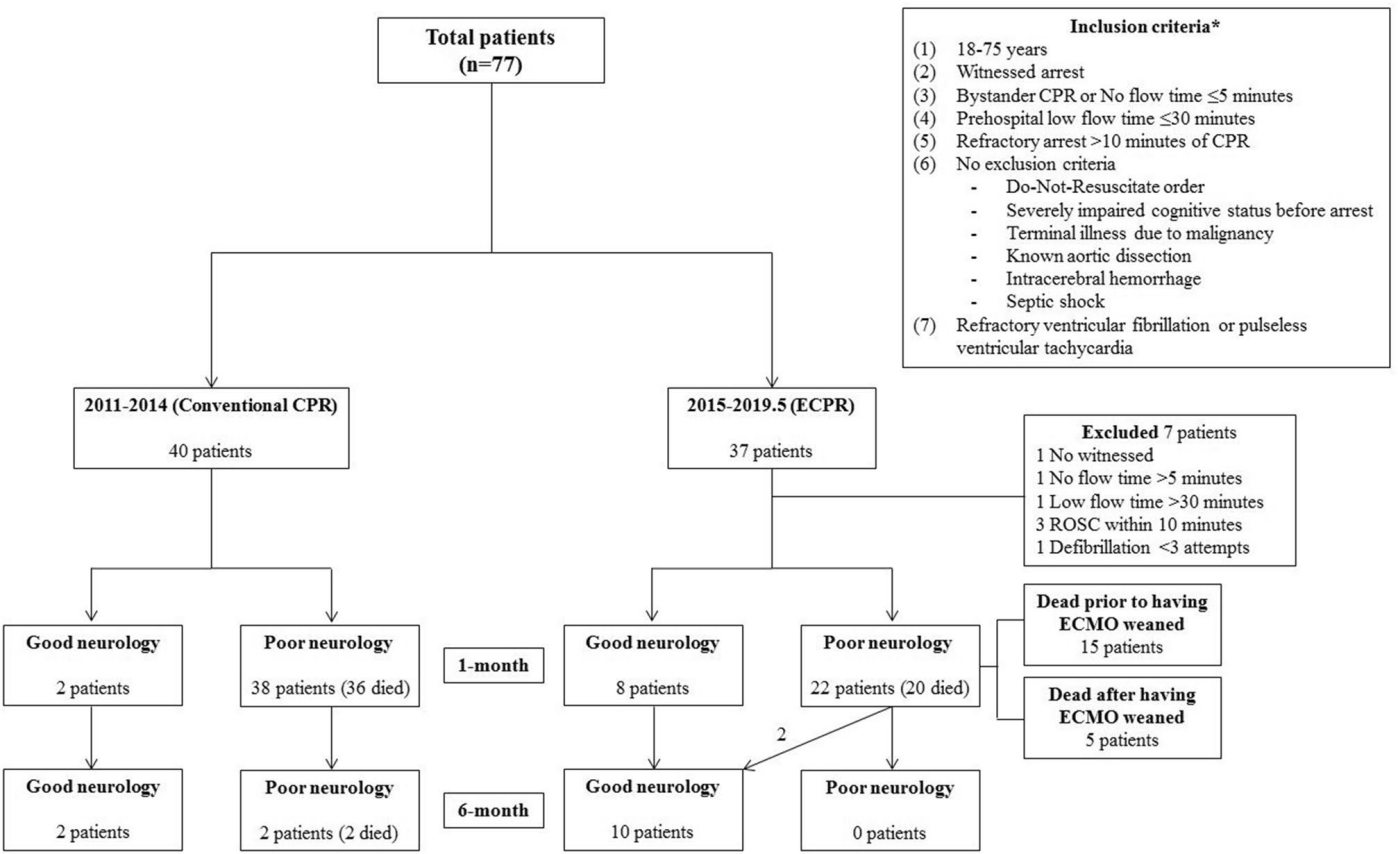
Flow diagram of patients included in our study. *All from (1) to (6) have to be met, or (7) has to be met. *CPR* cardiopulmonary resuscitation, *ECPR* extracorporeal cardiopulmonary resuscitation, *ROSC* return of spontaneous circulation, *ECMO* extracorporeal membrane oxygenation.

A total of 77 patients were enrolled at first from patients with OHCA from 2011 to May, 2019, in which 40 patients in “CCPR period” who met the inclusion criteria were included, 30 patients in “ECPR period” who received ECPR were included, but 7 patients in “ECPR period” were excluded due to lack of meeting the inclusion criteria. Exclusion specifics were as follows: 1 patient had no witness status, 1 patient had no-flow time > 5 min, 1 patient had low-flow time > 30 min, 1 patient had defibrillation attempts < 3, 3 patients were excluded due to ROSC within 10 min. The remaining 70 patients were analyzed in our study (Fig. 1).

The characteristics of the subjects, including age, sex, comorbidities, and CPR-related variables are listed by group, CCPR and ECPR, in Table 1. Age and the proportion of gender, comorbidities, witness status, bystander CPR, shockable rhythm initially documented as arrest rhythm, no-flow time, and low-flow time were not significantly different between the 2 groups.

**Table 1:** Characteristics of patients who met the inclusion criteria.

	Total patients (N = 70)	ECPR group (N = 30)	CCPR group (N = 40)	*P*-value
**Baseline characteristics**				
Age, years*	60 (52–68)	60 (55–67)	61 (51–70)	0.817
Male gender, N (%)	55 (78.6)	25 (83.3)	30 (75.0)	0.400
**Comorbidities, N (%)**				
Previous cardiac arrest	1 (1.4)	0 (0.0)	1 (2.5)	1.000
AMI	5 (7.1)	3 (10.0)	2 (5.0)	0.652
Angina	8 (11.4)	5 (16.7)	3 (7.5)	0.452
Previous PCI	5 (7.1)	3 (10.0)	2 (5.0)	0.652
Arrhythmia	2 (2.9)	2 (6.7)	0 (0.0)	0.203
Heart failure	6 (8.6)	3 (10.0)	3 (7.5)	1.000
Hypertension	27 (38.6)	13 (43.3)	14 (35.0)	0.715
Diabetes mellitus	15 (21.4)	6 (20.0)	9 (22.5)	0.629
Lung disease	7 (10.0)	1 (3.3)	6 (15.0)	0.116
Previous CVA	3 (4.3)	0 (0.0)	3 (7.5)	0.245
**OHCA circumstances, N (%)**				
Witness status				0.326
By EMS personnel	16 (22.8)	11 (36.6)	5 (12.5)	
By Layperson	53 (75.7)	18 (60.0)	35 (87.5)	
Bystander CPR	51 (72.9)	19 (63.3)	32 (80.0)	0.121
First documented arrest rhythm				
VF/pulseless VT	37 (52.9)	18 (60.0)	19 (47.5)	0.300
PEA	9 (12.9)	4 (13.3)	5 (12.5)	
Asystole	21 (30.0)	4 (13.3)	17 (42.5)	
Unidentifiable	12 (17.1)	4 (13.3)	8 (20.0)	
**Time from collapse to CPR termination, min**				
No flow time*	0 (0–3)	0 (0–4)	0 (0–2)	0.187
Low flow time	50.4 (15.48)	49.3 (16.7)	51.3 (14.6)	0.596
Pre-hospital CPR duration*	18 (12–23)	17 (7–23)	19 (16–22)	0.307
In-hospital CPR duration	33.3 (13.5)	33.6 (13.0)	33.1 (14.0)	0.885

*ECPR* extracorporeal cardiopulmonary resuscitation, *CCPR* conventional cardiopulmonary resuscitation, *AMI* acute myocardial infarction, *PCI* percutaneous coronary intervention, *CVA* cerebrovascular accident, *OHCA* out-of-hospital cardiac arrest, *CPR* cardiopulmonary resuscitation, *EMS* emergency medical services, *VF* ventricular fibrillation, *VT* ventricular tachycardia, *PEA* pulseless electric activity, *CPC* cerebral performance category.

*Median (IQR).

### Good neurologic outcomes at 6-months (primary outcome)

As for the primary outcome, of the ECPR group, 10 patients (33.3%) had a good neurologic status, which is 1 or 2 on CPC score, at 6-months post arrest, whereas only 2 patients (5.0%) patients treated with CCPR were found to have a good neurologic status (*P* = 0.002) (Table 2).

**Table 2:** Neurologic outcomes.

	Total patients (N = 70)	ECPR group (N = 30)	CCPR group (N = 40)	*P*-value
Survival to discharge, N (%)	16 (22.9)	10 (33.3)	6 (15.0)	0.071
**CPC at 1-month, N (%)**				
1	7 (10.0)	5 (16.7)	2 (5.0)	
2	3 (4.3)	3 (10.0)	0 (0.0)	
3	3 (4.3)	2 (6.7)	1 (2.5)	
4	1 (1.4)	0 (0.0)	1 (2.5)	
5	56 (80.0)	20 (66.7)	36 (90.0)	
Good neurologic outcome*	10 (14.3)	8 (26.7)	2 (5.0)	0.015
**CPC at 6-months, N (%)**				
1	7 (10.0)	5 (16.7)	2 (5.0)	
2	5 (7.1)	5 (16.7)	0 (0.0)	
3	0 (0.0)	0 (0.0)	0 (0.0)	
4	0 (0.0)	0 (0.0)	0 (0.0)	
5	58 (82.9)	20 (66.7)	38 (95.0)	
Good neurologic outcome*	12 (17.1)	10 (33.3)	2 (5.0)	0.002

*ECPR* extracorporeal cardiopulmonary resuscitation, *CCPR* conventional cardiopulmonary resuscitation, *CPC* cerebral performance category.

*CPC 1 or 2.

### Good neurologic outcome at 1-month and the rate of survival to discharge (secondary outcomes)

**Figure 2:**
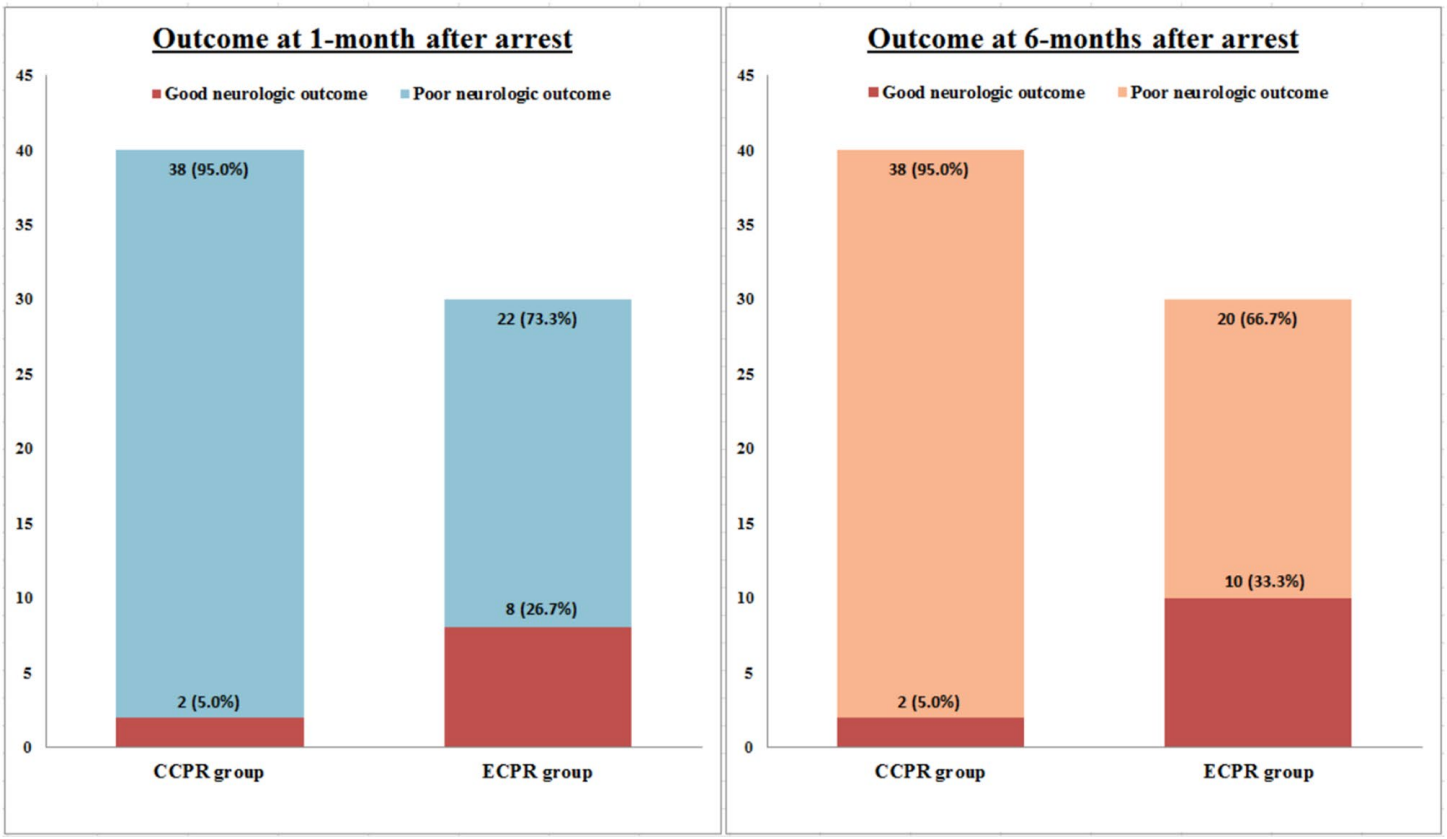
Good neurologic outcomes in patients receiving CCPR and ECPR. *CCPR* conventional cardiopulmonary resuscitation, *ECPR* extracorporeal cardiopulmonary resuscitation.

Similar results were found for good neurologic status at 1-month post arrest in 8 patients (26.7%) receiving ECPR and 2 patients (5.0%) receiving CCPR (*P* = 0.015). Comparing the primary outcome, 2 more patients who originally had poor neurologic outcome at 1-month gained good neurologic outcome at 6-months (Fig. 2).

**Figure 3:**
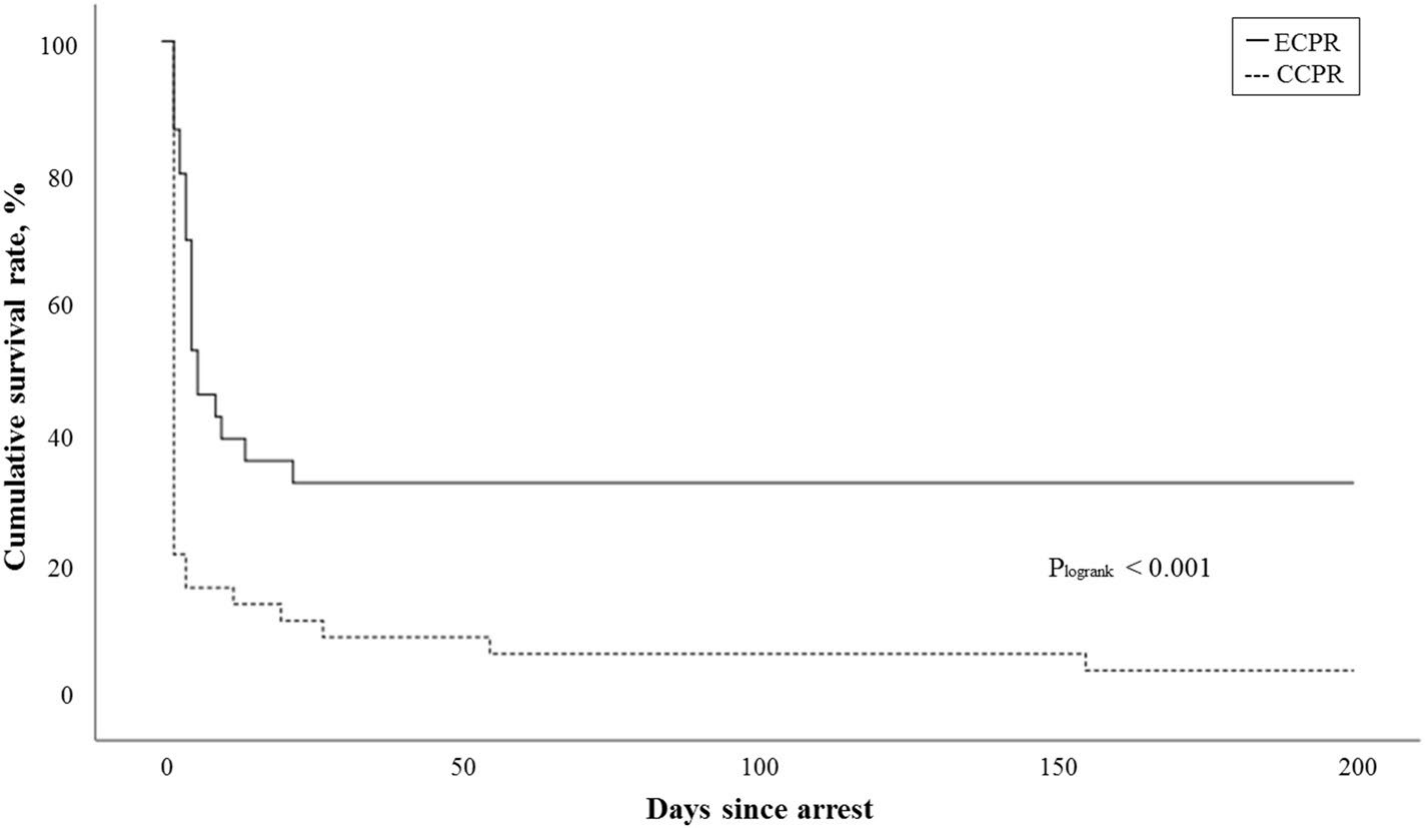
Survival rates in patients receiving CCPR and ECPR. *CCPR* conventional cardiopulmonary resuscitation, *ECPR* extracorporeal cardiopulmonary resuscitation.

The survival rate at the time of discharge among patients receiving ECPR (10 patients, 33.3%) was not significantly higher than that among those receiving CCPR (6 patients, 15.0%) (*P* = 0.071). However, using Kaplan–Meier curves to compare the cumulative survival rate, the survival rate at 6 months of patients receiving ECPR was significantly higher than that of those receiving CCPR by the log-rank test (*P* < 0.001) (Fig. 3).

Meanwhile, the rate of sustained ROSC (> 20 min) in the CCPR group was 27.5% (11/40), of whom 7 patients underwent targeted temperature management (TTM). The rates of TTM conducted did not have a significant difference between the ECPR and the CCPR group (73.3% vs 63.6%, P < 0.701).

### Complication of ECPR

Total 14 cases occurred when it comes to ECMO-associated complication (Table 3). Although there was no significant difference between the two groups, the group with good neurologic outcome at 6 months had only 2 different cases, which are bleeding and peripheral ischemia, and the patients suffered from these complications recovered and weaned ECMO successfully. On the other hand, in the group with poor neurologic outcome at 6 months, total 12 cases, which are 7 cases of bleeding, 3 of peripheral ischemia, and 2 of infection in the order of incidence, occurred. There was also 1 patient who died after recovering to 2 on CPC score in the hospital (Supplementary Table S1).

**Table 3:** Characteristics of patients included in “ECPR period”.

	Total patients (N = 30)	Good neurologic outcome at 6-months (N = 10)	Poor neurologic outcome at 6 months (N = 20)	P-value
**ECMO**				
Time from collapse to ECMO implementation, min	60.2 (18.9)	58.2 (26.4)	61.2 (14.5)	0.748
Duration on ECMO, h*	71.0 (39.0–116.0)	118.5 (76.0–203.0)	69.9 (29.5–81.8)	0.022
**ECMO-associated complication, N (%)**				0.100
Bleeding	8 (26.7)	1 (10.0)	7 (35.0)	
Peripheral ischemia	4 (13.3)	1 (10.0)	3 (15.0)	
Infection	2 (6.7)	0 (0.0)	2 (10.0)	
**CPR-related**				
No flow time, min*	0 (0–4)	0 (0–2)	0 (0–5)	0.055
Pre-hospital low flow time, min*	17 (7–23)	6 (0–16)	22 (11–26)	0.004
Total low flow time, min	49.3 (16.7)	37.3 (20.2)	55.3 (11.0)	0.005
Total epinephrine dose, mg	10.0 (4.0)	8.0 (5.0)	12.0 (3.0)	0.028
Number of defibrillation*	8.5 (5.0–12.0)	5.5 (0.0–8.0)	9.5 (5.5–12.0)	0.103
Presumed cardiac etiology, N (%)	29 (96.7)	10 (100.0)	19 (95.0)	1.000
VF/pulseless VT as initial rhythm, n (%)	18 (60.0)	6 (60.0)	12 (60.0)	1.000
Hospital LOS, day*	5 (3–20)	29 (18–85)	4 (2–7)	0.010
**Laboratory results**				
Initial lactate at presentation, mmol/L*	11.6 (9.2–14.7)	9.9 (7.3–15.0)	12.3 (9.7–14.7)	0.244
Initial pH	6.99 (0.17)	7.06 (0.15)	6.95 (0.17)	0.078
Troponin I, ng/mL*	0.140 (0.050–0.477)	0.090 (0.030–0.250)	0.217 (0.072–0.918)	0.285

*ECPR* extracorporeal cardiopulmonary resuscitation; *ECMO* extracorporeal membrane oxygenation, *CPR* cardiopulmonary resuscitation, *VF* ventricular fibrillation, *VT* ventricular tachycardia, *LOS* length of stay.

*Median (IQR).

### Subgroup analyses in patients with ECPR

Subgroup analyses were demonstrated in Table 3. Compared to the patients who had a poor neurologic status 6 months after arrest, those who had a good neurologic status showed longer ECMO duration and hospital stays, less use of epinephrine during CPR, and shorter prehospital and total low-flow time (all *P* < 0.05). However, time from collapse to ECMO implementation, ECMO-related complications, no-flow time, number of defibrillations, presence of shockable rhythm first documented as arrest rhythm, and initial laboratory results like lactate level, pH, and troponin I level, didn’t show significant difference between the 2 groups. Except for 1 patient, cardiac arrest of presumed cardiac origin occurred in 29 patients (96.7%).

## Discussion

Because ECPR is resource intensive and costly, it should be considered only in cases where the patient has a reasonably high likelihood of benefit and a potentially reversible illness. In this study we aimed to determine eligible criteria for applying ECPR in patients with OHCA by comparing 2 periods before and after ECMO implementation with the hope of increasing both good neurologic outcomes and overall survival rates and reducing unnecessary health cost. In our analyses we found that when applying the inclusion and exclusion criteria we set, the group who received ECPR had higher proportion of a good neurologic status at 6-months (33.3% vs 5.0%) and 1-month (26.7% vs 5.0%) in comparison with the group receiving CCPR. Likewise, overall survival rates at 6 months post presentation to the ED showed the same results as those of good neurologic status at 6 months post arrest (33.3% vs 5.0%). Of patients treated with ECPR, those who had good neurologic status at 6 months had shorter low-flow time, less use of epinephrine during CPR, and longer duration on ECMO and hospital stays compared with those with poor neurologic status.

In our study, we investigated both a long-term outcome as a good neurologic status at 6-months and a short-term outcome as a survival rate to discharge and a good neurologic status at 1-month. Patients in “ECPR period” compared with those in the “CCPR period” had both more favorable short- and long-term neurologic outcomes by 21.7% and 28.3%, respectively. Our results are consistent with the meta-analysis study by Kim et al.^16^, which found that applying a detailed indication of ECPR to patients with OHCA may increase the beneficial effect of ECPR. Also, our study showed higher overall survival rate than previous studies that reported a range of 4% to 36% in patients with adult OHCA^17–19^. Although advanced therapeutic methods like TTM have been implemented during the study period and there was a possibility for TTM to impact on the outcome, the rates of TTM conducted did not have a significant difference between the ECPR and the CCPR group in our study, implying that TTM did not have a major role specifically in the ECPR group. We can infer from our results that the criteria we hypothesized might have the potential to serve as a reference guideline for the initiation of ECPR in patients with OHCA.

In addition, in comparison with the previous study by Schober et al.^20^, which demonstrated good neurologic condition at 6-months in patients with ECPR, the long-term outcome of our study was higher (33.3% vs 14%) These discrepancies also might be explained by the presence of concrete inclusion criteria for initiation of ECPR. In their study, they included patients whose arrest was due to presumed cardiac origin and refractory despite 30 min of CCPR, not considering prehospital factors such as age and pre-arrest condition of patients. By applying a relatively broad-spectrum criteria and leaving the decision to initiate ECPR to physicians’ discretion, overall long-term outcomes turned out to be lower compared with those of our study. This suggests that more specific and well-organized criteria to apply ECPR for patients with OHCA increase its efficiency as well as good neurologic outcomes.

Lee et al.^21^ researched survival rates for patients whose ECPR was initiated by their own indication, which is similar to ours as well. The difference is that they didn’t limit age in their indication, so patients older than 75 years old were also included. Overall survival rates in their study, thus, differed from those in our study (18.9% vs 33.3%). In light of the study by Goto et al.^22^ advanced age was associated with lower survival rate in patients with ECPR, inclusion of patients older than 75 years old, which some previous studies contraindicated for ECPR^19,23^, might have affected the result to some extent. However, association between prognosis following ECPR and age has not been firmly established since a recent systematic review by Debaty et al.^24^ showed failure of their relationship. Therefore, as age should not be the sole barrier to applying ECPR in elderly patients when other criteria are met, range of age as an indication should be further investigated in the contemporary era because the elderly is only expected to grow.

As for no-flow time, Le Guen et al.^25^ showed its importance as a prognostic factor for patients with ECMO following OHCA, in which less than 5 min of no-flow time was associated with a good neurologic status for patients with OHCA who received ECMO. However, as shorter no-flow time doesn’t always mean the presence of bystander CPR, patients with less than 5 min of no-flow time who didn’t receive bystander CPR might be at risk of being excluded from ECMO application although there is a high chance of recovery with good neurologic status. To prevent missing these patients in the ED, we came up with inclusion criteria that cover both of them.

In subgroup analyses in our study, time from collapse to implantation of ECMO did not differ between groups with a good and a poor neurologic outcome (58.2 min vs 61.2 min). These results are similar to those of a previous study by Maekawa et al.^26^ and might have influenced the ECPR strategy of our hospital that physicians try to run ECMO within 60 min. However, according to the study by Yukawa et al.^27^, they demonstrated that the duration from cardiac arrest to initiation of ECMO is associated with a good neurologic outcome and showed that good neurologic outcomes decreased by 15% from 30% when the duration exceeded 40 min, which is inconsistent with the outcome in our study. This is thought to be the difference in the emergency medical services (EMS) system between 2 regions. Based on our regional EMS system, as ED physicians can rarely acquire detailed information about patients with OHCA before they arrive at the ED and EMS personnel are mostly not involved in Advanced Cardiovascular Life Support (ACLS), immediate implementation of ECMO right after patient arrival at the ED is difficult to be done in most cases. Although different healthcare setting might have led to discrepancies between the results, specific and detailed indication for the initiation of ECPR in our study is considered to be one of the reasons to attenuate the impact of the time from arrest to ECMO because they didn’t take patients’ pre-arrest medical illnesses into account in their indication as opposed to ours. However, prehospital low-flow time was significantly different between groups with good and poor neurologic outcomes. Assuming that the duration of implementing ECMO in the ED by skilled professionals is quite homogenous, it might be thought that the more important component that comprises low-flow time is prehospital low-flow time rather than total low-flow time. Although a study by Wengenmeyer et al.^28^ demonstrated that total low-flow time is strongly correlated with survival and mortality, prehospital low-flow time is considered to be the rate-limiting time of the total duration if application of ECMO in the ED is well trained given the assumption and our results. Therefore, prehospital low-flow time as the inclusion criteria rather than time from collapse to implementation of ECMO seems to be more appropriate in the well trained medical systems.

Complications related to ECMO should also be considered because bleeding, limb ischemia, or infection is common during its operation. In our study, bleeding was the most common complication (26.7%), followed by peripheral ischemia (13.3%) and infection (6.7%), which had lower rates of complication compared with studies by Sun et al.^29^ Most complications didn’t directly affect patient outcomes, but one case of septic shock was identified as a cause of death. Regarding this result, post-ECMO care is also important as a way to achieve overall good outcomes. As infection or bleeding at the cannulation sites or fasciotomy sites can lead severely vulnerable patients to dismal situations, standard guidelines to prevent these complications should be established. Overall, so called “extended chain of survival”, including an appropriate application of ECPR and post-ECMO care may help to achieve higher good outcomes.

There are several limitations to this study. First, this was a single-center study with a small size of patients included. Therefore, although we compared before-after study design with a quite balanced number of patients assigned into both groups, our results, especially the result of subgroup analyses, should be interpreted with caution as outcomes might differ in different healthcare settings. Regarding the limitation due to its before-and-after study design, our study might have been affected by bias such as Hawthorne effect, which physicians caring for the patients in the ECPR group treat and monitor more intensively. Nonetheless, considering that our primary endpoint was a good neurologic outcome which is less affected by this bias compared to the survival rate and it had been prohibited to withdraw life supporting therapies in Korea during our study period, this limitation is thought to less impact on our study. Second, CPC score may not reflect delicate neurologic function, such as memory or logical thinking, in spite of its widespread adoption to evaluate outcomes for patients with arrest. Since it is a crude measurement system and we evaluated based on the medical records or through telephone survey, it might have been possible to overestimate patients’ performance.

In conclusion, our study showed that application of the detailed indication, including (1) age (18 ~ 75 years), (2) witnessed arrest, (3) bystander CPR or no-flow time ≤ 5 min, (4) prehospital low-flow time ≤ 30 min, (5) refractory arrest > 10 min of CCPR at the ED, and (6) absence of exclusion criteria as well as refractory shockable rhythm before initiating ECPR could help to select the candidates for ECPR in a way to increase rates of neurologically intact survival in patients with OHCA and broaden its indication at the same time.

## Methods

### Data sources

This was a single-center before-after study which was conducted in the emergency department (ED) of an academic tertiary care hospital, which provides ECMO and PCI 24 h a day, 365 days a year in Seoul, Korea. To compare survival rates and good neurologic outcomes provided by ECPR with those of CCPR, we analyzed 2 separate periods. The first period, from 2011 to 2014, was defined as the “CCPR period,” and the second period, between 2015 and May of 2019, was referred to as the “ECPR period”. The eligibility of the ECMO indication itself was prospectively evaluated. Outcomes of patients treated with ECMO (ECPR group) were compared with patients of a historical control (CCPR group). All experiments were performed in accordance with relevant named guidelines and regulations. The study protocol was approved by the institutional review board of Asan Medical Center, which waived the requirement to obtain informed consent.

### Inclusion and exclusion criteria

The inclusion criteria were decided based on the previous studies and the real-world practice for initiating ECPR, in which many physicians do ECPR to patients with refractory OHCA although they do not fully meet commonly adopted inclusion criteria, such as age ≤ 75 years, witnessed arrest^15,24^. Given these findings, the inclusion criteria that we set are as follows; (1) patients aged between 18 and 75 years whose OHCA was witnessed by a bystander, (2) bystander CPR or no-flow time ≤ 5 min, (3) prehospital low-flow time ≤ 30 min, (4) refractory arrest > 10 min of CCPR at the ED, and (5) absence of exclusion criteria. To cover patients with ventricular fibrillation, who are traditionally considered to be candidates for ECPR, we also included patients who had refractory ventricular fibrillation or pulseless ventricular tachycardia, which are defined as those who are resistant to at least 3 defibrillation attempts, 300 mg of amiodarone, and do not acquire return of spontaneous circulation (ROSC) in spite of > 10-min attempts of CCPR even if they didn’t meet the inclusion criteria^30–32^. We excluded patients whose Do-Not-Resuscitate order was placed before the arrest, or who had severely impaired cognitive status before the arrest, terminal illness due to malignancy, known aortic dissection, intracerebral hemorrhage, and septic shock.

### Study population

In the first period (CCPR period), patients with OHCA who met the inclusion criteria we set, but did not have ECPR were selected. And in the second period (ECPR period), patients with OHCA who had ECPR were enrolled and patients who didn’t meet the criteria were excluded. Medical information and data from electronic medical records which were reviewed in a retrospective manner included demographic information like age and gender, comorbidities, witness status, presence of bystander CPR, initially documented arrest rhythm, prehospital no-flow time, low-flow time including prehospital and in-hospital, respectively, survival rates at discharge, and outcomes at 1-month and 6-months since their presentation. When there was no documentation of patient information in the medical records, we processed them as missing values.

### Study endpoint

Primary outcome was a good neurologic status, defined as 1 or 2 on the Cerebral Performance Category (CPC) score, which is able to complete activities of daily living with moderate impairment or less at 6-months post presentation to the ED on day of arrest^33^. Secondary outcomes were defined as a good neurologic status during the 1-month period after arrest and survival rate at the time of discharge. Good neurologic outcome and survival rate between OHCA patients who were treated with ECPR and patients who met the ECPR criteria and underwent CCPR were compared. Subgroup analyses were done among patients who received ECPR based on neurologic status at 6-months post arrest. Time from collapse to ECMO implementation, ECMO duration, and proportion of ECMO-associated complications, Advanced Cardiovascular Life Support (ACLS)-related values, such as total epinephrine dose, number of defibrillations, and laboratory values like initial pH, lactate level, and troponin I level were investigated.

### Statistical analysis

Continuous variables were expressed as the mean value with standard deviation for normal distribution and compared using the Student’s t-test. Variables with a non-normal distribution were expressed as medians with their interquartile range (IQR) and analyzed by the Mann–Whitney test. Categorical variables were demonstrated as proportions and compared by the Chi-squared test or Fisher’s exact test, as appropriate. Cumulative survival rates are presented with Kaplan–Meier curves for the groups, and the differences were tested using the log-rank test. Subgroup analyses were conducted in the same methods described above after dichotomizing groups into good (CPC 1 or 2) and poor (CPC 3–5) neurologic outcomes. All statistical analyses were done using IBM SPSS Statistics V21.0 (SPSS Inc., Chicago, IL). *P* < 0.05 was considered statistically significant.

## Supplementary Information

Supplementary Information.
