# Supplementary material for: Promising candidates for extracorporeal cardiopulmonary resuscitation for out-of-hospital cardiac arrest
Source: Sci Rep. 2020 Dec 17;10:22180. doi: 10.1038/s41598-020-79283-1 (PMC7746692; doi:10.1038/s41598-020-79283-1)
Supplement: Supplementary file 1 — Supplementary Information. [file 41598_2020_79283_MOESM1_ESM.docx]

**Promising candidates for extracorporeal cardiopulmonary resuscitation for out-of-hospital cardiac arrest**

Yo Sep Shin, MD; Youn-Jung Kim, MD, PhD; Seung Mok Ryoo, MD, PhD; Chang Hwan Sohn, MD, PhD; Shin Ahn, MD, PhD; Dong Woo Seo, MD, PhD; Won Young Kim, MD, PhD

Department of Emergency Medicine, Asan Medical Center, University of Ulsan College of Medicine, Seoul, Korea

**Corresponding author:**

Won Young Kim, MD, PhD

Department of Emergency Medicine, Asan Medical Center, University of Ulsan College of Medicine

88 Olympic-ro 43-gil, Songpa-gu, Seoul 05505, Korea

Tel: 82-2-3010-3350

Fax: 82-2-3010-3360

Email: [wonpia73@naver.com](mailto:wonpia73@naver.com)

**Supplementary table 1.** Characteristics of the patient who deteriorated after being recovered

| Age / Gender | Presumed etiology of arrest | Past medical history | No flow time | Prehospital low flow time | ECMO duration | Complication of ECMO |
| --- | --- | --- | --- | --- | --- | --- |
| 46 / Male | Cardiac | None | 4 min | 30 min | 211hr | - Bleeding  - Peripheral ischemia  - Infection |
| Hospital course | | | | | | |
| HD 01 | 2-vessel disease was found by CAG and PCI was done at proximal RCA and LAD. | | | | | |
| HD 04 | Fasciotomy was done at right calf due to compartment syndrome caused by bleeding. | | | | | |
| HD 10 | ECMO was weaned as cardiac function was recovered. | | | | | |
| HD 13 | ET tube was removed and mental status was recovered to being alert. | | | | | |
| HD 19 | Septic shock due to CRAB bacteremia occurred presumably by infection at fasciotomy site. | | | | | |
| HD 21 | Expired due to septic shock. | | | | | |

*CAG* coronary angiography; *PCI* percutaneous coronary intervention; *RCA* right coronary artery; *LAD* left anterior descending coronary artery; *ECMO* extracorporeal membrane oxygenation; *HD* hospital day; *CRAB* ; carbapenem-resistant Actinobacter baumannii
